# The role of circulating tumor cells in shaping the future of early-stage lung cancer treatment: a clinical point of view

**DOI:** 10.1016/j.jlb.2025.100309

**Published:** 2025-07-06

**Authors:** Filippo Lococo, Jessica Evangelista, Carolina Sassorossi, Elisa De Paolis, Annalisa Campanella, Maryam Heydary, Yaqun Liu, Davide Brechot, Patrizia Paterlini, Stefano Margaritora, Angelo Minucci

**Affiliations:** a Thoracic Surgery Unit, Università Cattolica del Sacro Cuore, Largo F.Vito 1, Rome, Italy; b Thoracic Surgery Unit, Fondazione Policlinico Universitario A. Gemelli IRCCS, Rome, Italy; c Departmental Unit of Molecular and Genomic Diagnostics, Genomics Core Facility, Gemelli Science and Technology Park (G-STeP), Fondazione Policlinico Universitario A. Gemelli IRCCS, Rome, 00168, Italy; d Clinical Chemistry, Biochemistry and Molecular Biology Operations (UOC), Fondazione Policlinico Universitario A. Gemelli IRCCS, Rome, Italy; e Rarecells, Faculté de Médecine Necker Enfants Malades, Paris, France

Recently, Bahmaie and co-workers reported an insightful review [1] exploring the diagnostic and prognostic roles of circulating tumor cells (CTCs) in oncology. The Authors concluded that further investigations and an interdisciplinary collaboration are needed to address the current gaps in the strategies of cancer management.

Inspired by these conclusions, we would like to contribute to the debate on such a hot topic by focusing on early-stage non-small cell lung cancer (NSCLC) setting from a clinical point of view.

The management of lung cancer is becoming increasingly complex due to the growing influence of molecular data and the rapid development of target therapies.

The use of "liquid biopsy" has already become a clinical reality in metastatic disease and is expected to be increasingly adopted in clinical practice for all stages, including screening of populations at risk [2]. Most of the available literature focuses on ctDNA, with only preliminary results reported so far in early-stage NSCLC [2].

On the other hand, several research teams using different techniques have proposed the isolation of CTCs, as reported in Ref. [1]. However, CTC methods face experimental criticisms regarding cell viability and vulnerability, as well as technical challenges related to the efficiency of CTCs enrichment. Therefore, there is an urgent need for effective isolation methods of CTC.

In this setting, the ISET® (Isolation by SizE of Tumor cells) technology already reported for isolating CTCs from NSCLC blood samples [4] was not reported in Ref. [1]. The isolation method [3] relies on the larger size of CTCs compared to the majority of leukocytes, increasing the recovery of CTCs. ISET® has two possible readouts, allowing both cytopathological and molecular identification of CTCs.

Nowadays, this method has been explored as a complementary tool to a tissue biopsy or monitoring the effect of targeted therapies in advanced NSCLC-patients [4]. On the other hand, the ISET®-method with cytological readout has also been proposed as diagnostic and prognostic tool in operable NSCLC [5]. In this pilot study performed on 75 NSCLC-patients before surgery (52 Stage I-II patients) and 10 healthy volunteers, the CTCs detection was 60 % in patients, while no CTCs were found in lung cancer-free donors. The Authors identified single CTCs (sCTC) in 46 % of patients, and heterotypic clusters of CTCs and leukocytes (hetCLU) in 31 %. While they observed that the prevalence of both sCTC and hetCLU may potentially predict the risk of disease recurrence within the cohort of early-stage or advanced stage the limited number of subjects did not allow to draw any definitive conclusion.

In this setting, our research team is conducting a prospective trial enrolling 50 early-stage surgically-resected NSCLC-patients with baseline preoperative and longitudinal blood sample collection every 3 months (for Stage II/III) or every 6 months (for Stage I).Blood samples (20 ml) will be used for CTC-DNA extraction and analysis following ISET®, as well as for ctDNA analysis.

The study was approved by our local ethical committee (Lazio Area 3, 25-07-2024, num°6948). The objectives are to compare the sensitivity and accuracy of these two methods and to evaluate the potential of CTC-DNA by ISET® as a viable biomarker for minimal residual disease in predicting tumor recurrence. We would expect to analyze CTC-DNA in a small but significant percentage of early-stage lung cancers and to evaluate its prognostic impact in order to detect a viable biomarker for tailoring neo/adjuvant treatments.

We would greatly appreciate the Authors’ reflections on the points raised.

## Declaration of competing interest

The authors declare the following financial interests/personal relationships which may be considered as potential competing interests: Lococo Filippo reports financial support was provided by University Hospital Agostino Gemelli. If there are other authors, they declare that they have no known competing financial interests or personal relationships that could have appeared to influence the work reported in this paper.
